# Antimicrobial susceptibilities and comparative whole genome analysis of two isolates of the probiotic bacterium *Lactiplantibacillus plantarum,* strain ATCC 202195

**DOI:** 10.1038/s41598-021-94997-6

**Published:** 2021-08-05

**Authors:** Lisa G. Pell, Rachael G. Horne, Stuart Huntley, Hafizur Rahman, Sanchita Kar, Mohammad Shahidul Islam, Kara C. Evans, Samir K. Saha, Aaron Campigotto, Shaun K. Morris, Daniel E. Roth, Philip M. Sherman

**Affiliations:** 1 Centre for Global Child Health, Hospital for Sick Children, Toronto, ON Canada; 2 Cell Biology Program, Research Institute, Hospital for Sick Children, Toronto, ON Canada; 3 International Flavors & Fragrances Inc., Madison, WI USA; 4 Child Health Research Foundation, Dhaka, Bangladesh; 5 Department of Paediatrics, Faculty of Medicine, University of Toronto, Toronto, ON Canada; 6 Division of Microbiology, Hospital for Sick Children, Toronto, ON Canada; 7 Dalla Lana School of Public Health, University of Toronto, Toronto, ON Canada; 8 Division of Infectious Diseases, Hospital for Sick Children, Toronto, ON Canada; 9 Gastroenterology, Hepatology and Nutrition, Hospital for Sick Children, 555 University Avenue, Toronto, ON M5G 1X8 Canada; 10 Paediatric Medicine and Child Health Evaluative Sciences, Hospital for Sick Children, Peter Gilgan Centre for Research and Learning, 686 Bay Street, Toronto, ON M5G 0A4 Canada

**Keywords:** Genome, Genomics

## Abstract

A synbiotic containing *Lactiplantibacillus plantarum* [American Type Culture Collection (ATCC) strain identifier 202195] and fructooligosaccharide was reported to reduce the risk of sepsis in young infants in rural India. Here, the whole genome of two isolates of *L. plantarum* ATCC 202195, which were deposited to the ATCC approximately 20 years apart, were sequenced and analyzed to verify their taxonomic and strain-level identities, identify potential antimicrobial resistant genes and virulence factors, and identify genetic characteristics that may explain the observed clinical effects of *L. plantarum* ATCC 202195. Minimum inhibitory concentrations for selected antimicrobial agents were determined using broth dilution and gradient strip diffusion techniques. The two *L. plantarum* ATCC 202195 isolates were genetically identical with only three high-quality single nucleotides polymorphisms identified, and with an average nucleotide identity of 99.99%. In contrast to previously published reports, this study determined that each isolate contained two putative plasmids. No concerning acquired or transferable antimicrobial resistance genes or virulence factors were identified. Both isolates were sensitive to several clinically important antibiotics including penicillin, ampicillin and gentamicin, but resistant to vancomycin. Genes involved in stress response, cellular adhesion, carbohydrate metabolism and vitamin biosynthesis are consistent with features of probiotic organisms.

## Introduction

Accumulating evidence supports the use of probiotics to prevent morbidity and mortality during early infancy in both high- and low- to middle-income settings^1–3^. Despite marked heterogeneity in probiotic strains studied across randomized controlled trials, recent evidence has sparked interest in the potential of *Lactiplantibacillus plantarum* ATCC 202195^4^, formerly named *Lactobacillus plantarum* ATCC 202195, to improve early infant health outcomes, particularly in resource-constrained settings^5^.

*Lactiplantibacillus plantarum* ATCC 202195 is a Gram-positive, non-spore forming, facultative anaerobe originally isolated from human feces^6^. Oral administration of *L. plantarum* ATCC 202195 plus fructooligosaccharide (FOS) to newborns has been shown to lead to sustained intestinal colonization in early infancy^5,7^. In a placebo-controlled randomized trial in rural India, oral administration of *L. plantarum* ATCC 202195 plus FOS to newborns (birthweight $\ge$ 2000 g and gestational age $\ge$ 35 weeks) was reported to reduce episodes of clinical sepsis within the first 60 days of life, compared to placebo, by 40% (95% CI 0.48–0.74)^5^. A 78% (95% CI 0.09–0.53) reduction in the incidence of blood culture-confirmed bacterial sepsis was also reported in the same study^5^. Lactobacilli were not grown in any blood culture, and adverse events involving the gastrointestinal tract were similar between trial arms, with the investigational product being generally well tolerated^5^. The direction and magnitude of the estimated effect sizes, combined with early safety data, indicate that *L. plantarum* ATCC 202195 has the potential to improve infant health outcomes, especially in settings where the burden of severe infection and rates of infant mortality are high.

Prior to the aforementioned clinical trial, an isolate of *L. plantarum* ATCC 202195, which we refer to herein as *L. plantarum* ATCC 202195-A, was deposited to the American Type Culture Collection (ATCC) in January 1999 as a patent submission deposit (U.S. Patent Number: 6,132,710)^6^ and assigned the ATCC number 202195. Notably, the patent holder permitted select commercial sale of the isolate; however, no rights to propagate or characterize the isolate were permitted. Approximately twenty years after the initial patent deposit to the ATCC, a new isolate of the *L. plantarum* ATCC 202195 strain was submitted to the ATCC, which we refer to here as *L. plantarum* ATCC 202195-B. Commercial production and distribution by the ATCC were authorized by the patent holder. At the time of both submissions to the ATCC, whole genome sequence data were not publicly available for either *L. plantarum* ATCC 202195-A or *L. plantarum* ATCC 202195-B. Thus, the strain-level identity of both isolates was unknown and the identity of *L. plantarum* ATCC 202195-B was not genetically verified.

Strain-level identification ensures that microbes held in culture repositories are correctly identified and handled^8^, provides a genetic signature to monitor both probiotic engraftment and probiotic-associated bacteremia, and provides a baseline sequence to assess whether the genetic composition of the strain changes over time. ATCC, as an International Depository Authority, assumes no liability for the accuracy of the depositors’ information concerning patent deposits. Since probiotics are living microorganisms, as part of their natural evolutionary process they may acquire deleterious genetic mutations that could mitigate proposed health benefits^9,10^, which are often strain-specific^11,12^. Thus, verifying the genetic identity of a probiotic strain prior to its administration and throughout the manufacturing process is of paramount importance.

Despite increasing clinical interest in *L. plantarum* ATCC 202195, there is only one peer-reviewed published report describing the draft genome of this probiotic strain^13^, and one additional complete genome assembly released on Genbank (accession GCA_010586945.1), with no linked peer reviewed report. Neither genome addressed a critical concern regarding the genetic lineage of *L. plantarum* ATCC 202195.

The antimicrobial susceptibility profile of a probiotic strain and the potential for the ability to transfer antimicrobial resistance (AMR) genes or virulence factors to other microorganisms is a key consideration in determining a probiotic’s suitability for use in humans and/or animals. There is general consensus that antimicrobial susceptibility profiles should be determined for all probiotics^14^ and that a microbe must be confirmed to contain no acquired or transferrable AMR^15^ or virulence factors^16^ prior to its use as a probiotic agent. The need to determine the antimicrobial susceptibility profile of a probiotic strain extends beyond the potential to transfer AMR elements to other microorganisms. If a probiotic is deemed to be the cause or suspected cause of bacteremia or other invasive infection, knowledge of AMR patterns can inform clinical treatment considerations. In addition, given that there is growing evidence in support of the combined delivery of probiotics and antibiotics^17,18^, knowledge of the AMR phenotype of a microbe could inform the development of novel multi-component treatment regimens.

While the antimicrobial susceptibility profile of *L. plantarum* ATCC 202195-A was previously reported and it was indicated not to harbor a plasmid^5^, minimum inhibitory concentrations (MICs) for this probiotic strain have not been described nor have there been sequence-based reports to corroborate resistance phenotypes.

In the present study, we aimed to determine the sequence of the bacterial genome and verify the taxonomic and strain-level identify of *L. plantarum* ATCC 202195-A and *L. plantarum* ATCC 202195-B. By analyzing the bacterial genome, we aimed to identify AMR genes and potential virulence factors, and to provide possible mechanistic insights underlying the clinical effects of *L. plantarum* ATCC 202195. We also aimed to determine the MICs for each isolate of *L. plantarum* ATCC 202195 by employing a panel of 20 clinically relevant antimicrobial agents. Together, these findings can be used to inform the design of future clinical studies employing *L. plantarum* ATCC 202195 either as a probiotic or in a synbiotic formulation.

## Results

### Antimicrobial susceptibility and minimum inhibitory concentrations of *L. plantarum* ATCC 202195-A and *L. plantarum* ATCC 202195-B

Based on broth dilution assays, the antimicrobial susceptibility profiles of *L. plantarum* ATCC 202195-A and *L. plantarum* ATCC 202195-B were identical (Table 1). Both isolates were found to be sensitive to penicillin (MIC = 4 µg/ml), ampicillin (MIC = 2 µg/ml), meropenem (MIC $\le$ 0.25 µg/ml), clindamycin (MIC $\le$ 0.12 µg/ml), linezolid (MIC = 4 µg/ml), erythromycin (MIC $\le$ 0.25 µg/ml), chloramphenicol (MIC = 8 µg/ml), gentamicin (MIC $\le$ 2 µg/ml), piperacillin/tazobactam (MIC $\le$ 8 µg/ml) and daptomycin (MIC $\le$ 0.25 µg/ml). By contrast, both isolates were resistant to vancomycin (MIC $\ge$ 256 µg/ml) and tetracycline (MIC $\ge$ 32 µg/ml). MICs for ceftriaxone (MIC $\le$ 0.5 µg/ml), levofloxacin (MIC $\ge$ 8 µg/ml), ciprofloxacin (MIC $\ge$ 4 µg/ml), quinupristin/dalfopristin (MIC = 2 µg/ml), rifampin (MIC $\ge$ 8 µg/ml), trimethoprim/sulfamethoxazole (MIC $\le$ 0.5/9.5 µg/ml), gatifloxacin (MIC = 2 µg/ml) and oxacillin (MIC = 4 µg/ml) were determined; however, clinical breakpoints for these antimicrobials and *Lactobacillus *spp. are not available from the CLSI^19^, EUCAST^20^, or EFSA^21^. Where clinical breakpoints were available, susceptibility interpretations were identical for all antimicrobials assessed by both broth dilution and E-testing, except for tetracycline (Supplementary Table 1). The MIC for tetracycline as determined by broth dilution ($\ge$ 32 µg/ml) was four times higher than the value determined by E-testing (8 µg/ml) for *L. plantarum* ATCC 202195-A. In microbroth dilution assays, the concentration of tetracycline only went up to 16 µg/ml (MIC reported as equal to or greater than two-times the antibiotic concentration in the well where growth occurred). The range of tetracycline concentrations evaluated by E-testing was 0.016–256.0 µg/ml and growth inhibition intersected the side of the E-strip at 8 µg/ml (Supplementary Table 1). Thus, the true MIC value for *L. plantarum* ATCC 202195 and tetracycline may lie in between the values described by microbroth dilution assays and E-testing. MICs determined by E-tests were identical (penicillin, erythromycin and vancomycin) or within one doubling dilution (ceftriaxone (MIC = 1 µg/ml), chloramphenicol (4 µg/ml) and gentamicin (1 µg/ml)) to those determined by broth dilution assays for six of the eight antimicrobials assayed (Table 1). The MIC for ciprofloxacin ($\ge$ 32 µg/ml) as determined by E-testing was 8 times higher than the value determined using the broth dilution assay, which likely reflects the antibiotic concentration range tested in each assay. *L. plantarum* ATCC 202195-B was negative for beta-lactamase activity.

**Table 1:** Antimicrobial susceptibility and minimum inhibitory concentrations of *L. plantarum* ATCC 202195-A and *L. plantarum* ATCC 202195-B.

Antibiotic class	Antimicrobial	Broth dilution MICs and interpretations			Prior Evidence from *L. plantarum* ATCC 202195, other strains of *L. plantarum*, and other types of lactobacilli
		*L. plantarum* ATCC 201295-A	*L. plantarum* ATCC 202195-B	Interpretation	
		MIC (µg/ml)	MIC (µg/ml)		
Beta-lactam: Penicillin	Penicillin	4	4	S^a^ –^bc^	Clinical breakpoint interpretation consistent with previous disc diffusion findings for the *L. plantarum* ATCC 202195 strain^5^ Observed MIC value within one doubling dilution of the upper end of the MIC range that was previously reported for 46 other isolates of *L. plantarum* (0.5–2 mg/l)^22^
	Ampicillin	2	2	S^a,b,c^	Strain-specific susceptibility data were not previously available for ampicillin Observed MIC value within the range of values previously reported for 46 other strains of *L. plantarum* (0.125–2 mg/l)^22^ and 10 other *L. plantarum* strains (0.5–32 µg/ml)^23^
	Oxacillin + 2% NaCl	4	4	–^a,b,c^	Strain-specific susceptibility data were not previously available for oxacillin. Oxacillin activity against other strains of *L. plantarum* has not previously been reported
Beta-lactam: Carbapenem	Meropenem	≤ 0.25	≤ 0.25	S^a,c^ –^b^	Strain-specific susceptibility data were not previously available for meropenem Meropenem activity against other strains of *L. plantarum* has not previously been reported
Beta-lactam: Cephalosporin	Ceftriaxone	≤ 0.5	≤ 0.5	–^a,b,c^	Previous disc diffusion assays suggest that *L. plantarum* ATCC 202195 is ceftriaxone-sensitive5. Clinical breakpoint interpretations were not available for ceftriaxone and lactobacilli Ceftriaxone activity against other strains of *L. plantarum* has not previously been reported
Beta-lactam: Penicillin/Beta-lactamase inhibitor	Piperacillin/tazobactam	≤ 8	≤ 8	S^c^ –^a,b^	Strain-specific susceptibility data were not previously available for piperacillin/tazobactam Piperacillin/tazobactam activity against other strains of *L. plantarum* have not been described previously
Lincosamide	Clindamycin	≤ 0.12	≤ 0.12	S^a,b,c^	Strain-specific susceptibility data were not previously available MIC value was within the range of values independently reported by two different groups for 46 (0.032–1 mg/L)^22^ and 10 (0.03–32 µg/ml)^23^ other *L. plantarum* strains
Oxazolidinone	Linezolid	4	4	S^a^ –^b,c^	Strain-specific susceptibility data were not previously available MIC value was within the range of values previously reported for 10 other strains of *L. plantarum* (2–8 µg/ml)^23^ and within one doubling dilution of the upper end of the range observed for 46 other *L. plantarum* strains (1–2 mg/l)^22^
Macrolide	Erythromycin	≤ 0.25	≤ 0.25	S^a,b^ IE^c^	Strain-specific susceptibility data were not previously available MIC value was within the range of values independently reported by two different groups for 10 (0.25–16 µg/ml)^23^ and 46 (0.016–0.5 mg/l)^22^ other strains of *L. plantarum*
Glycopeptide	Vancomycin	≥ 256	≥ 256	R^a,c^ –^b^	Interpretation consistent with previous disc diffusion findings for the *L. plantarum* ATCC 202195 strain^5^
Chloramphenicol	Chloramphenicol	8	8	S^b,c^ –^a^	Strain-specific susceptibility data were not previously available MIC was within the range of values previously reported for 46 other strains of *L. plantarum* (2–8 mg/l)^22^ and 10 other strains of *L. plantarum* (4-16 µg/ml)^23^
Fluoroquinolone	Levofloxacin	≥ 8	≥ 8	–^a,b,c^	Strain-specific susceptibility data were not previously available Levofloxacin activity against other strains of *L. plantarum* has not previously been reported
	Ciprofloxacin	≥ 4	≥ 4	–^a,b,c^	Using disc diffusion assays, it was previously reported that *L. plantarum* ATCC 202195 is ciprofloxacin-resistant^5^ Clinical breakpoints were not available to interpret MICs for ciprofloxacin and *L. plantarum* Among ten *L. plantarum* type strains previously tested for susceptibility to ciprofloxacin, the MIC values ranged between 16 and 256 µg/ml^23^. The range of ciprofloxacin concentrations tested by microbroth dilution only went to as high as 2 µg/ml. By contrast, the range of ciprofloxacin concentrations evaluated by E-testing was 0.002–32 µg/ml. The true MIC value for *L. plantarum* ATCC 202195 and ciprofloxacin may be closer to the value described by E-testing (i.e., ≥ 32 µg/ml)
	Gatifloxacin	2	2	–^a,b,c^	Strain-specific susceptibility data were not previously available Gatifloxacin activity against other strains of *L. plantarum* has not previously been reported
Aminoglycoside	Gentamicin	≤ 2	≤ 2	S^b^ –^a,c^	Interpretation is consistent with previous disc diffusion findings for *L. plantarum* ATCC 202195^5^ MIC value fell within the range published independently by two different research groups for 46 ($\le$ 1–8 mg/l)^22^ and 10 other *L. plantarum* (0.5–512 µg/ml)^23^ isolates
Tetracycline	Tetracycline	≥ 32	≥ 32	R^b^ –^a,c^	Strain-specific susceptibility data were not previously available for tetracycline MIC value within the range of values previously reported for 10 other strains of *L. plantarum* (4 and 64 µg/ml)^23^
Macrolide-lincosamide-streptogramin	Quinu/Dalfopristin	2	2	–^a,b,c^	Strain-specific susceptibility data were not previously available MIC of quinupristin/dalfopristin for *L. plantarum* ATCC 202195 was within the range reported for 10 other *L. plantarum* strains (1–8 µg/ml)^23^ and within one doubling dilution of the upper end of the MIC range reported for 46 other *L. plantarum* isolates (0.063–1 mg/L)^22^
Lipopeptide	Daptomycin	≤ 0.25	≤ 0.25	S^a^ –^b,c^	Strain-specific susceptibility data were not previously available Daptomycin activity against other strains of *L. plantarum* have not been described previously
Rifamycin	Rifampin	≥ 8	≥ 8	–^a,b,c^	Strain-specific susceptibility data were not previously available MIC of rifampin was within one doubling dilution of the upper end of the MIC range reported for 10 other *L. plantarum* strains (0.12–4 µg/ml)^23^
Sulfonamide	Trimethoprim/Sulfamethoxazole	≤ 0.5/9.5	≤ 0.5/9.5	–^a,b,c^	Strain-specific susceptibility data were not previously available MIC of trimethoprim/sulfamethoxazole was within the range of MIC values previously reported for 46 other strains of *L. plantarum* (0.5– > 512 mg/l)^22^

Clinical breakpoint interpretations were categorized as susceptible ("S"), intermediate ("I"), resistant ("R"), no interpretive criteria available ("–”), and insufficient evidence (“IE”).

^a^Interpretation based on CLSI, ^b^EFSA, and on ^c^EUCAST.

### Comparisons of the *L. plantarum* ATCC 202195-A and *L. plantarum* ATCC 202195-B genomes

**Figure 1:**
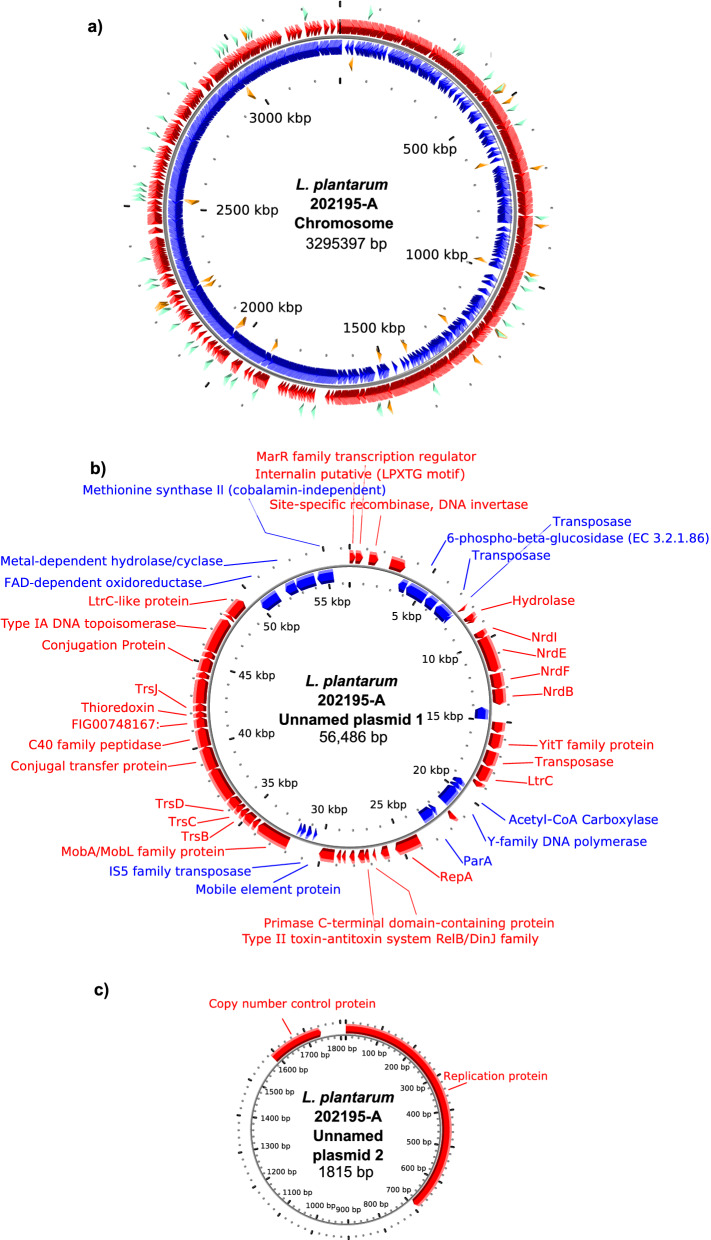
Circular genomic map of the *L. plantarum* ATCC 2021295-A. (**a**) The chromosome is 3,295,397 base pairs in size and has a G + C content of 44%. (**b**) Unnamed plasmid 1 (56,486 base pairs, G + C content = 40%); and, (**c**) Unnamed plasmid 2 (1815 base pairs, G + C content = 37.4%). Images were generated using CGViewer^24^ and gene annotation was prepared using RASTtk^25^. Forward strand genes are denoted as red arrows, reverse strand genes are denoted as blue arrows, RNA genes are denoted as orange arrows, and repeat regions are denoted as aqua coloured arrows. The original image was generated using GC Viewer (https://paulstothard.github.io/cgview/) and the text was modified in Inkscape v1.0.1 (https://inkscape.org/release/inkscape-1.0.1/).

To corroborate our phenotypic data, the genomes of both isolates were sequenced and compared. The genomic sequence for isolate *L. plantarum* ATCC 202195-A was generated by International Flavors & Fragrances Inc (IFF) (formerly Dupont Nutrition & Biosciences) from DNA extracted from pure monoculture purchased from ATCC in 2017. The resulting sequencing reads assembled into a complete genome containing one chromosome (3,295,397 bp) (Fig. 1a) and two plasmids; unnamed plasmid 1 (56,486 bp) (Fig. 1b) and unnamed plasmid 2 (1815 bp) (Fig. 1b), respectively, with a total genome length of 3,353,698 base pairs (G + C content = 44.3%). The genomic sequences for *L. plantarum* ATCC 202195-B were generated using an ATCC isolate purchased in 2019 by The Hospital for Sick Children, (Toronto, ON) and reads were assembled into a draft genome with a total of 146 contigs, with 66 contigs > 150 bp, and a total genome length of 3,332,603 base pairs. There was a difference in genome length of 21,098 base pairs relative to *L. plantarum* ATCC 202195-A (G + C content = 44.3%). Two plasmids were also present in the draft genome of *L. plantarum* ATCC 202195-B. All of the sequencing reads from *L. plantarum* ATCC 202195-B mapped to the genome of *L. plantarum* ATCC 202095-A, with coverage of greater than 1000×, indicating that this discrepancy in genome length is likely a result of variation in sequence methodology and genome assemblers.

Comparison between the two bacterial genomes for single nucleotide polymorphisms (SNPs) identified just three high-quality variants (Table 2). Two of the three variants were localized within one gene encoding a putative surface-layer protein (LPXTG motif), one of which was a missense mutation and the other was a stop-gain mutation. The remaining SNP was located within a transposase, IS4 family gene and was classified as a deletion variant (Table 2).

**Table 2:** Summary of single nucleotide variants between *L. plantarum* ATCC 202195-A and *L. plantarum* ATCC 202195 -B.

Nucleotide position	Variation (LP202195-A/LP202195-B)	Quality score^a^	Sequence depth at variant site	Strand allele probability (SAP)^b^	Strand reference probability (SRP)^c^	Putative function	SNP type
2978657	CAATG/CATG	217.678	12	29.068	0	Transposase, IS4 family	Deletion
3220411	G/A	158.242	102	31.2394	174.557	Surface protein (LPXTG motif)	Nonsynonymous
3219413	G/T	118.453	667	825.998	376.504	Surface protein (LPXTG motif)	Nonsynonymous

^a^Based on an estimated probability that the alternate allele is present at the loci, as calculated using Freebayes^26^.

^b^Strand balance probability for the alternate allele, as calculated using Freebayes^26^.

^c^Strand balance probability for the reference genome, as calculated using Freebayes^26^.

**Figure 2:**
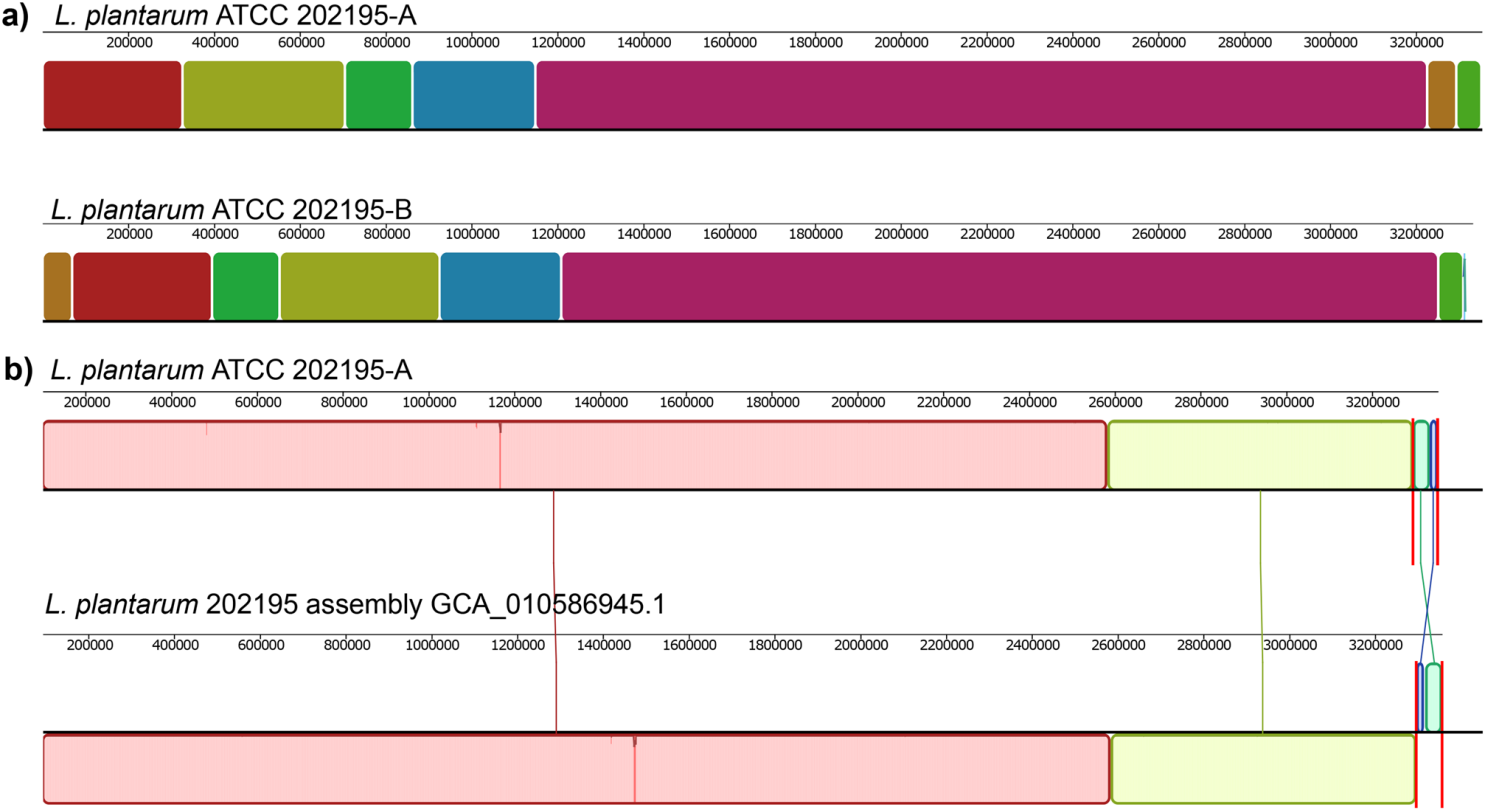
Comparison of sequenced isolates of *L. plantarum* ATCC 202195 (**a**) Graphical representation of a global sequence alignment of the co-linear blocks (identified using progressiveMAUVE^27^) conserved between the *L. plantarum* ATCC 2021295-A and *L. plantarum* ATCC 2021295-B genomes. (**b**) Mauve alignment of *L. plantarum* ATCC 202195-A and *Lactiplantibacillus plantarum* ATCC 202195 assembly GCA_010586945.1. Matching coloured blocks correspond to co-linear blocks shared between the sequences. The original image was generated by Mauve software (http://darlinglab.org/mauve/user-guide/mauvealigner.html) and edited in Inkscape v1.0.1 (https://inkscape.org/release/inkscape-1.0.1/).

**Figure 3:**
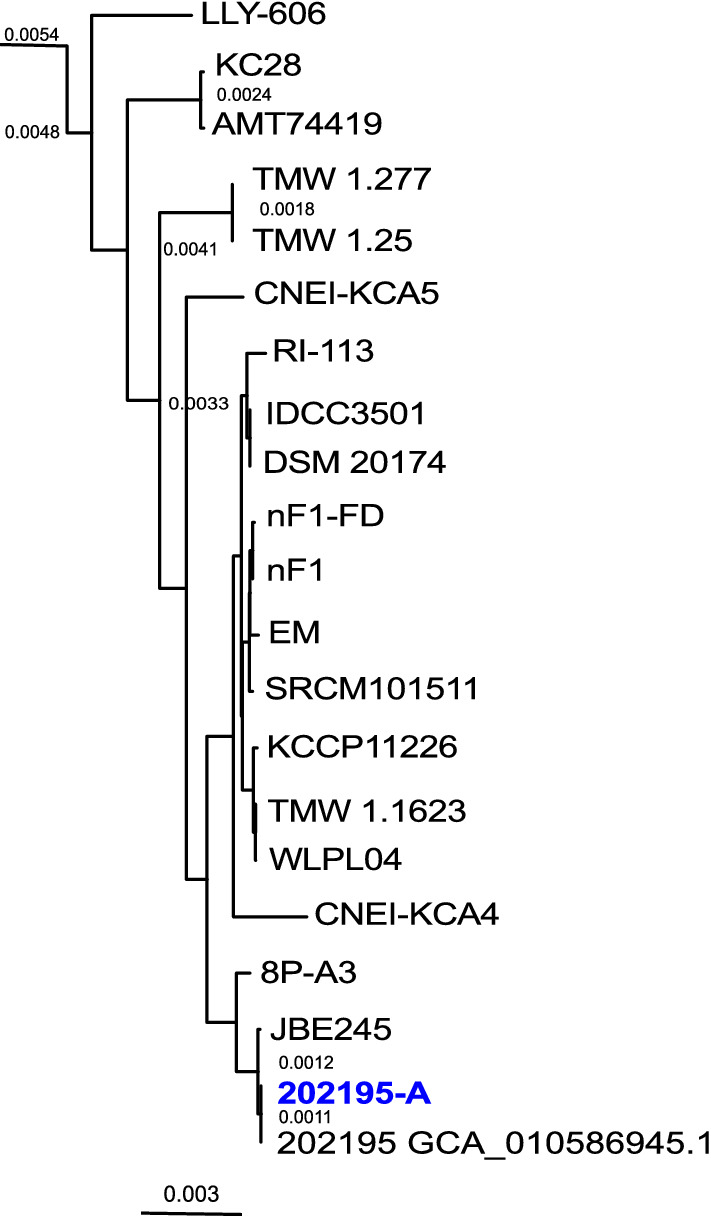
Phylogenetic tree of the clade containing *L. plantarum* ATCC 202195-A. A phylogenetic tree was generated using only the core genome alignment that was derived from the pan-genome analysis of 133 *L. plantarum* strains. *L. plantarum* ATCC 202195 is shown in blue and *L. plantarum* ATCC 202195 assembly GCA_010586945.1 is labelled as '202195 GCA_010586945.1'. Branch lengths equal to or greater than 0.0011 are shown. The complete phylogenetic tree of all 133 *L. plantarum* strains can be found in Supplementary Fig. 1. The phylogenetic tree was generated by Figtree (http://tree.bio.ed.ac.uk/stats.html) and edited in Inkscape v1.0.1 (https://inkscape.org/release/inkscape-1.0.1/).

The average nucleotide identity (ANI) between the two genomes was 99.99% and global alignment identified 10 locally colinear blocks with a minimum weight of 109 (Fig. 2a). Comparing the genome of *L. plantarum* ATCC 202195-A with a previously released complete genome assembly for *L. plantarum* strain ATCC 202195 (accession number: GCA_010586945.1) resulted in an ANI of 99.99% and global alignment identified four locally colinear blocks with a minimum weight of 19,941 (Fig. 2b). In a comparison of our complete genome of *L. plantarum* ATCC 202195-A with the partial draft *L. plantarum* ATCC 202195 genome released by Wright et al.^13^ (accession number: GCA_004354995.1), we found an ANI of 99.98%. Although Wright et al. did not report any associated plasmid sequences, we found 100% sequence homology between both plasmids described here with contigs 10 and contigs 16 of the GCA_004354995.1 genome assembly. *L. plantarum* ATCC 202195 complete genome assembly GCA_010586945.1 was identified as the closest genetic relative to *L. plantarum* ATCC 202195-A (Fig. 3), followed by *L. plantarum strain* JBE245. *L. plantarum* ATCC 202195 genome assembly GCA_010586945.1 lacked sequence homology to unnamed plasmid 2 that was identified in both *L. plantarum* ATCC 202195-A and *L. plantarum* ATCC 202195-B.

### Antimicrobial resistance and virulence genes

As genomic comparison revealed near identical genome sequences, screening for AMR and virulence genes was limited to the most complete genome (*L. plantarum* ATCC 202195-A). Initial high stringency screening of AMR databases did not identify potential antimicrobial resistance genes. By reducing the screening threshold stringency, three partial matches to the AMR genes *LmrD*, *LmrC* and *rpoB* from CARD^28^ and 12 partial matches to known virulence factors in VFDB^29^ were identified (Table 3). Based on observed phenotypic resistance patterns, additional targeted screening was performed against known resistance genes for vancomycin (*ddl*), tetracycline [*tet*(M), *tet*(S), *tet*(W), *tet*(O), and *tet*(Q)], and ciprofloxacin (*gyrA*) (Table 3). *L. plantarum* ATCC 202195-A genome was found to contain an intrinsic active site mutation (F261Y) in the *ddl* gene that confers resistance to vancomycin. A single partial match to a tetracycline resistance gene, *tetM*, was found, and while *gyrA* gene was found to be present in *L. plantarum* ATCC 202195-A genome, no mutations responsible for conferring fluoroquinolone resistance were identified.

**Table 3:** Antimicrobial resistance genes and putative virulence genes present in the genomes of *L. plantarum* ATCC 2021295-A and *L. plantarum* ATCC 202195-B.

Class	Gene	Product	*L. plantarum* ATCC 202195-A	
			Coverage (%)	Identity (%)
Virulence genes^a^	*cpsI*	UDP-galactopyranose mutase	92.81	67.28
	*clpC*	endopeptidase Clp ATP-binding chain C	27.28	69.19
	*clpP*	ATP-dependent Clp protease proteolytic subunit	93.47	70.54
	*hasC*	UDP-glucose pyrophosphorylase	87.65	71.81
	*htpB*	Hsp60 60 K heat shock protein HtpB	90.74	64.8
	*cpsJ*	ABC transporter ATP-binding protein	41.15	66.67
	*lap*	Listeria adhesion protein Lap	48.83	64.35
	*bsh*	bile salt hydrolase	89.98	71.9
	*cpsA*	undecaprenyl diphosphate synthase	33.33	74.45
	*clfA*	Clumping factor A fibrinogen-binding protein	31.26	77.7
	*clpE*	ATP-dependent protease	65.7	66.67
	*cap8P*	capsular polysaccharide synthesis enzyme	25	75.68
Antimicrobial resistance genes	*lmrD*^b^	Multidrug transporter subunit	31.07	67.41
	*rpoB*^b^	beta subunit of RNA polymerase	15.42	67.92
	*lmrC*^b^	Multidrug transporter subunit	41	67.57
	*tetM*^c^	Tetracycline resistance protein	95	30.76

^a^Virulence genes were identified by comparing DNA sequences from both isolates to the Virulence Factor Database (VFDB)^29^.

^b^Antimicrobial resistance gene were identified using ABRicate^30^ and The Comprehensive Antibiotic Resistance Database (CARD)^28^, and initial results were verified by BLASTn^31^.

^c^*L. plantarum* ATCC 202195 gene loci G9282_00305 compared to KRM38573.1 *L. aviarius* subsp. aviarius DSM 20655.

### Genome features of *L. plantarum* ATCC 202195-A

Annotation of the genome, including two putative plasmids, using the RASTtk pipeline revealed a total of 3286 coding sequences, including 2287 and 999 sequences assigned as functional and hypothetical proteins, respectively. A total of 72 transfer RNA genes and 16 ribosomal RNA genes were identified. Unnamed plasmid 1 likely represents a conjugative plasmid with a total of 56,486 base pairs (Fig. 1b) that encode 64 genes, 50 of which have assigned putative functions. Unnamed plasmid 2, a non-conjugative plasmid (Fig. 1c), contains just 2 genes: one gene encodes a putative replication protein and the other codes for a copy number control protein. Evaluating the assembled plasmid sequences for sequence homology revealed an almost exact match of unnamed plasmid 2 with that of *Pediococcus claussenii* ATCC BAA-344 plasmid pPECL-1^32^ (sequence identity of 99%, and 100% coverage by BLASTn). Unnamed plasmid 1 has high homology to the unnamed plasmid (60,765 base pair in size) identified in the previously released *L. plantarum* ATCC 202195 genome assembly GCA_010586945.1 (92% query coverage and 100% identity). Neither plasmid contains genes that code for identified AMR or a virulence factor. Notably, unnamed plasmid 1 encodes several genes that upon transfer would be advantageous to the recipient, including a manganese transport protein, MntH, magnesium and cobalt transport protein, CorA, 6-phospho-beta-glucosidase (EC 3.2.1.86), BglA-2, adenosylhomocysteinase (EC 3.3.1.1) and methionine synthase II (cobalamin-independent).

### Functional annotation of *L. plantarum* ATCC 202195-A genome

**Figure 4:**
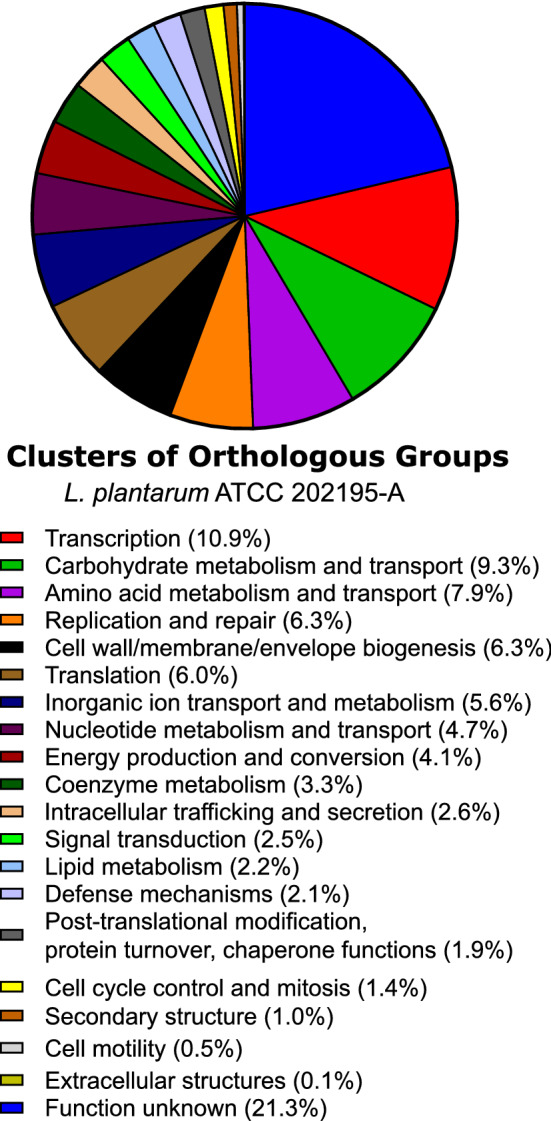
Functional categorization of 2860 predicted open reading frames (ORFs) in the genome of *L. plantarum* ATCC 202195-A based on Clusters of Orthologous Groups of proteins (COGs). Image was generated in Graphpad 9.

Using the eggNOG database^33^, 2860 genes were assigned to at least one of the cluster orthologous group families comprising 20 functional groups. Of those 2860 genes, 10.9% were assigned to the transcription functional group, followed by 9.3% of genes assigned to carbohydrate transport and metabolism, and 7.8% of genes assigned to amino acid transport and metabolism (Fig. 4). Further functional characterization by KEGG Pathways analysis, revealed a similar functional distribution of genes, with the majority of identified pathways attributed to nucleotide, carbohydrate and amino acid metabolism. A PATRIC subsystem analysis revealed that 32% of identified genes from *L. plantarum* ATCC 202195-A mapped to 10 of the 11 possible superclasses, including metabolism (38.4%), protein processing (14.9%), and stress response, defence and virulence (9.3%).

### Stress response and adhesion

Based on subsystem analyses, a total of 33 unique genes were sub-categorized as stress response (Table 4). The *L. plantarum* ATCC 202195-A genome encodes genes involved in acid tolerance including four bile salt acid hydrolases (*bsh1, bsh2, bsh3* and *bsh4*), and eight sodium-proton antiport genes. A total of 16 genes were subclassified as stress response genes, including nine genes that encode putative universal stress response proteins and seven genes that play a role in the oxidative stress response including glutathione peroxidase, NADH peroxidase and catalase. Several genes responsible for responses to heat and cold stress were also identified, including a number of putative protein-folding chaperones within the well characterized Clp protein family (*clpC, clpP, clpL, clpX, clpB* and *clpE*), and four homologous members of the cold shock protein family (*cspP*, *cspL*, *cspR* and *cspC*). Two of the three small heat shock proteins (sHSP) genes encoded by the *L. plantarum* reference strain WCFS1^34^ were also identified in *L. plantarum* ATCC 202195-A.

**Table 4:** Stress response and defense features in the genome of *L. plantarum* ATCC 202195-A.

Sub-classification	Subsystem name	Gene count	Role count
Stress response	Glutathione: Redox cycle	2	2
	Universal stress protein family	9	1
	Cluster containing Glutathione synthetase	2	2
	Hydroxy-fatty acid production as stress response	1	1
	Glutathione: Biosynthesis and gamma-glutamyl cycle	1	1
	Protection from Reactive Oxygen Species	1	1
Stress response: heat/cold shock	Cold shock proteins of CSP family	4	1
	Heat shock dnaK gene cluster extended	14	12
Stress response: osmotic stress	Osmoregulation	6	2
	Choline uptake and conversion to betaine clusters	7	8
Undefined	Hfl operon	2	2

Subsystems analysis of the superclasses Stress Response, Defense, and Virulence, was performed using The Pathosystems Resource Integration Center (PATRIC)^35^, and Rapid Annotations Using Subsystems Technology (RASTtk)^25^ for annotations.

The *L. plantarum* ATCC 202195-A genome also contains several genes that facilitate cellular adhesion, including two genes that encode fibronectin binding proteins, one gene that encodes a mucin binding protein, two mucus adhesion promoting protein genes (*mapA*), two enolases, two surface layer LPXTG anchored proteins and four putative LPXTG internallins.

### Carbohydrate processing and utilization of fructo-oligosaccharides (FOS)

Of the 266 genes encoded by *L. plantarum* ATCC 202195-A that were identified as being involved in carbohydrate metabolism according to COG annotation, only 94 of these genes were annotated as carbohydrate-active enzymes by the CAZy database^36^. Specifically, *L. plantarum* ATCC 202195-A was found to encode 52 glycoside hydrolases, 35 glycosyltransferases, three carbohydrate binding modules, two carbohydrate esterases and two auxiliary activity enzymes, indicating a strong metabolic capability to degrade and process complex carbohydrates. Embedded within the pool of carbohydrate processing genes was a conserved pts1BCA operon^37^, which is responsible for the import of short-chain FOS (scFOS) into the cytosol. The *L. plantarum* ATCC 202195-A genome also contains three gene clusters critical to the production of the short-chain fatty acid butyrate from acetyl-CoA.

### Biosynthesis of B complex vitamins

Evaluating the *L. plantarum* ATCC 202195-A genome for genes involved in the biosynthesis of B vitamins, revealed a complete gene cluster (*folA, folB, folC1, folC2, folD, folE, folK, folP* and *folQ*) that is involved in folate (vitamin B9) biosynthesis and utilization (Table 5). In contrast to *L. plantarum* strain WCFS1^38^, the *L. plantarum* ATCC 202195-A genome contains a complete riboflavin operon, including the *ribA, ribB, ribH, ribE* and *ribG* genes, required for riboflavin biosynthesis (Table 5). *L. plantarum* ATCC 202195-A also encodes genes involved in thiamine and biotin utilization and salvage; however, based on the genome sequences, the microbe appears incapable of de novo synthesis of either of these vitamins.

**Table 5:** List of putative genes in the *L. plantarum* ATCC 202195-A genome involved in B vitamin biosynthesis.

B vitamins	Gene symbol	Gene product
Folate (B9)	–	Substrate-specific component FolT of folate ECF transporter
	–	Substrate-specific component FolT of folate ECF transporter
	*folB*	Dihydroneopterin aldolase (EC 4.1.2.25)
	*folK*	2-Amino-4-hydroxy-6-hydroxymethyldihydropteridine pyrophosphokinase (EC 2.7.6.3)
	*folE*	GTP cyclohydrolase I (EC 3.5.4.16) type 1
	*folC2*	Dihydrofolate synthase (EC 6.3.2.12) at Folylpolyglutamate synthase (EC 6.3.2.17)
	*folP*	Dihydropteroate synthase (EC 2.5.1.15)
	–	Dihydrofolate reductase (EC 1.5.1.3)
	–	Dihydrofolate reductase (EC 1.5.1.3)
	*ecfT*	Transmembrane component of general energizing module of ECF transporters
	*ecfA2*	ATPase component of general energizing module of ECF transporters
	*ecfA*	ATPase component of general energizing module of ECF transporters
	*tdk*	Thymidine kinase (EC 2.7.1.21)
	*glyA*	Serine hydroxymethyltransferase (EC 2.1.2.1)
	*folC*	Dihydrofolate synthase (EC 6.3.2.12) at Folylpolyglutamate synthase (EC 6.3.2.17)
	*thyA*	Thymidylate synthase (EC 2.1.1.45)
	*folA*	Dihydrofolate reductase (EC 1.5.1.3)
	*trmFO*	Methylenetetrahydrofolate–tRNA-(uracil-5-)-methyltransferase TrmFO (EC 2.1.1.74)
	*fhs*	Formate–tetrahydrofolate ligase (EC 6.3.4.3)
	fmt	Methionyl-tRNA formyltransferase (EC 2.1.2.9)
	*folD*	Methenyltetrahydrofolate cyclohydrolase (EC 3.5.4.9)/Methylenetetrahydrofolate dehydrogenase (NADP+) (EC 1.5.1.5)
	*fthC*	5-Formyltetrahydrofolate cyclo-ligase (EC 6.3.3.2)
	*metK*	S-Adenosylmethionine synthetase (EC 2.5.1.6)
	–	Substrate-specific component FolT of folate ECF transporter
Riboflavin (B2)	*ribC1*	FMN adenylyltransferase (EC 2.7.7.2)/Riboflavin kinase (EC 2.7.1.26)
	*ribF*	MN adenylyltransferase (EC 2.7.7.2)/Riboflavin kinase (EC 2.7.1.26)
	*ribA*	3,4-Dihydroxy-2-butanone 4-phosphate synthase (EC 4.1.99.12) / GTP cyclohydrolase II (EC 3.5.4.25)
	*ribG*	Diaminohydroxyphosphoribosylaminopyrimidine deaminase (EC 3.5.4.26)/5-amino-6-(5-phosphoribosylamino)uracil reductase (EC 1.1.1.193)
	*ribD*	Putative FMN hydrolase (EC 3.1.3.-); 5-Amino-6-(5′-phosphoribitylamino)uracil phosphatase
	*ribT*	COG0454 Histone acetyltransferase HPA2 and related acetyltransferases
	*ribH*	6,7-Dimethyl-8-ribityllumazine synthase (EC 2.5.1.78)
	*npp*	Alkaline phosphodiesterase I (EC 3.1.4.1)/Nucleotide pyrophosphatase (EC 3.6.1.9)
	*ribE*	Riboflavin synthase (EC 2.5.1.9)

### Bacteriocins

The *L. plantarum* ATCC 202195-A genome encodes three plantaricin specific operons, including the regulator operon *plnABCD,* the bacteriocin operon *plnEFI* and the transport operon *plnGHTUVW*, which are required to produce the class IIb bacteriocins, plantaricin plnEF and plnA. Bacteriocins are a heterogeneous group of bioactive bacterial peptides that act as antimicrobial agents against closely related susceptible bacterial species^39^. Similar to the reference strain *L. plantarum* WCFS1^38^, *L. plantarum* ATCC 202195-A also encodes the plantaricin immunity protein *plnL*, directly upstream from the regulatory operon.

## Discussion

Prior to the widespread use of a probiotic agent in humans, it is imperative to delineate microbial susceptibility to antimicrobial agents, strain level identification and establish if antimicrobial resistance is intrinsic or has the potential to be transferred to other microorganisms^40^. Here, we present the first comparative genome analysis of two isolates of *L. plantarum* ATCC 202195, which were procured from separate ATCC deposits that occurred approximately 20 years apart. Our analysis revealed previously unreported features of the *L. plantarum* ATCC 202195 genome including two putative plasmid sequences. In addition, we have provided the most complete antimicrobial susceptibility characterization of this clinically important strain to date.

To be considered safe for human consumption, it has been suggested that probiotics should be susceptible to at least two major classes of currently available antibiotics^41^. While transferable resistance is uncommon among lactic acid-producing bacteria, acquired antibiotic resistance has been identified in isolates considered for probiotic or nutritional uses^22^. Herein, we evaluated the genomic sequence of *L. plantarum* ATCC 202195 for the presence of AMR genes and tested the susceptibility of each isolate against a panel of 20 antimicrobial agents across 16 classes of antibiotics and corroborated these findings with the genome sequence data. The two isolates had identical in vitro antimicrobial susceptibility profiles, were both found to be sensitive to eight of the newly tested antimicrobials (Table 1), and with two exceptions (rifampin and penicillin), the observed MICs fell within the range of values previously reported for other strains of *L. plantarum*^22,23^. MICs observed for rifampin and penicillin were both within one doubling dilution of the upper range of MICs previously reported for other *L. plantarum* isolates^23^.

Screening the genome of both *L. plantarum* ATCC 202195 isolates for sequence homology to AMR genes revealed only partial hits, with low sequence identity and coverage, to the multidrug efflux heterodimers *LmrCD* and *rpoB* (β-subunit of RNA polymerase) for which a mutation is known to confer resistance to rifampin^22,23^. In vitro susceptibility testing for rifampin using a broth dilution assay demonstrated growth at the highest rifampin concentration tested, which may be supportive of rifampin resistance; however, we were unable to make a conclusive determination in this regard due to the lack of established clinical breakpoints. Additionally, a partial match to a tetracycline resistance gene *tetM*, was identified which may be responsible for the phenotypic resistance observed in this strain. Both isolates were resistant to vancomycin, consistent with previously published findings for this strain generated by disc diffusion assay^42^. The signature active site mutation in the *ddl* gene, which confers resistance to vancomycin, was conserved within the *L. plantarum* ATCC 202195 genome, corroborating our phenotypic findings. While a clinical breakpoint interpretation was not available for ciprofloxacin activity against lactobacilli, previous disc diffusion assays with *L. plantarum* ATCC 202195 suggested that the strain is resistant to ciprofloxacin^5^. Herein, we report ciprofloxacin MICs suggestive of resistance for *L. plantarum* ATCC 202195 using the broth dilution and E-testing assays, respectively. Notably, while the target *gyrA* gene was present in the *L. plantarum* ATCC 202195 genome, no fluoroquinolone resistant mutations were identified in this gene and thus, ciprofloxacin resistance may likely be attributed to efflux mechanisms. Both isolates tested in the present study were sensitive to penicillin (MIC = 4 µg/ml) and gentamicin (MIC $\le$ 2 µg/ml), consistent with previous findings for *L. plantarum* ATCC 202195 obtained using disc diffusion^5^. Since the World Health Organization currently recommends the use of ampicillin plus gentamicin for the initial empiric treatment of neonates with suspected sepsis^43^, evidence that *L. plantarum* ATCC 202195 is susceptible to these agents further supports the safety of this probiotic strain in the unlikely event that an infant administered *L. plantarum* ATCC 202195 were to develop probiotic-related bacteremia or infection.

Verifying the genetic lineage of these two isolates is of critical importance to any future clinical work using this probiotic strain. Herein, our comparative genome analyses revealed that *L. plantarum* ATCC 202195-A and *L. plantarum* ATCC 202195-B are identical with only three high-quality SNPs identified between the two genomes, and an ANI of 99.99%. Relative to other strains of *L. plantarum*, *L. plantarum* ATCC 202195 forms a distinct branch on the phylogenetic tree and its closest phylogenetic neighbours were *L. plantarum* ATCC 202195 (GCA_010586945.1) and *L. plantarum* JBE245, a food-related bacterium^44^. In contrast to previous reports, which indicated that *L. plantarum* ATCC 202195 did not contain a plasmid^5,13^, we identified two putative plasmids in both isolates of *L. plantarum* ATCC 202195; however, these plasmids were only assembled into complete sequences in the *L. plantarum* ATCC 202195-A genome.

Plasmid sequence assembly and identification is challenging due to the presence of repeat sequences and the use of short reads for genome sequencing^45^. Herein, we resolved plasmid sequences by combining whole genome sequencing data with sequences that were generated using DNA obtained from a plasmid extraction. Often, plasmid sequences identified in *L. plantarum* strains do not encode any genes that functionally impact the host^46^. However, we annotated several plasmid-related genes that encode potentially impactful biological properties, including metal transport, amino acid synthesis and carbohydrate processing. Specifically, BglA-2 (6-phospho-beta-glucosidase) encoded on unnamed plasmid 1 is responsible for catalyzing the conversion of cellbiose-6P to glucose and glucose-6P, which then can enter the glycolic pathway to generate further energy for the cell^47^. Transfer of this gene through a conjugative plasmid could potentially provide an adaptive advantage to the plasmid recipient microbe^47^.

Evaluating the functional attributes of a probiotic strain by annotation of the complete genome sequence sheds light onto mechanistic underpinnings of documented clinical outcomes. Previous work has found that oral administration of *L. plantarum* ATCC 202195 leads to sustained colonization^5^. Here we report strain specific functional gene annotation related to clinical phenotypic findings. Notably, the *L. plantarum* ATCC 202195 genome was found to contain an array of genes involved in environmental stress responses, including genes related to cellular adhesion, bile acid tolerance, and responses to heat and cold stress, as well as oxidative stress. Since bile acids have known antimicrobial properties^48^, it is advantageous to the bacterium that *L. plantarum* ATCC 202195 encodes several bile acid hydrolase genes, the products of which can metabolize conjugated bile salts^49^. The *L. plantarum* ATCC 202195 genome also contains the heat shock protein genes: *clpE*, *clpX* and *clpP*. The production of the ATPases ClpE and ClpX and the ClpP protease increase in response to heat-shock and other microenvironmental stresses^50^, and play an important role in maintaining protein quality by regulating proteolysis^51^.

Microbial production of secondary metabolites is a potential mechanism by which probiotics can improve host health. Microbial production of the short-chain fatty acid butyrate has been linked to enhanced intestinal epithelial barrier function^52^, modulation of inflammatory status^52,53^, regulation of colonic T-cell differentiation^54^ and overall homeostasis in the intestinal tract. Multiple studies have linked the production of short-chain fatty acids to the utilization of prebiotics, including fructo-oligosaccharides and galacto-oligosaccharides^55^. The *L. plantarum* ATCC 202195 genome encodes three clusters of genes involved in butyrate production, as well as an operon for fructo-oligosaccharide metabolism. In other strains of *L. plantarum*, butyrate production is upregulated in the presence of fructo-oligosaccharides^56,57^. A potential relationship between the metabolism of fructo-oligosaccharides and the production of butyric acid by *L. plantarum* ATCC 202195 is intriguing, since FOS could well have played a role in the positive clinical outcomes in newborns reported by Panigrahi et al.^5^ Moreover, the identification of a diverse repertoire of carbohydrate active enzymes suggests that *L. plantarum* ATCC 202195 is able to degrade, utilize and synthesize both simple and complex saccharides. The functional annotation of the complete genome of *L. plantarum* ATCC 202195 provides the foundation for future mechanistic investigations; in vitro and in vivo studies are needed to verify the impact of each highlighted functional characteristic on the microbe and host.

Advances in next generation sequencing have increased the availability of high-quality genomic data and improved our ability to identify phylogenetic relationships; however, technical differences in genome sequencing protocols and genome assembly tools can result in artifactual variance^58^. Here, we sequenced, assembled and compared genomes of two isolates from the same strain, *L. plantarum* ATCC 202195. Our methodology used a combination of short- and long-read sequences for ATCC 202195-A genome assembly and only short-read sequences for ATCC 202195-B, as well as two different assembly tools. To limit technical bias in our comparative analyses, we compared unassembled sequencing reads from *L. plantarum* ATCC 202195-B against the complete assembled *L. plantarum* ATCC 202195-A genome, and utilizing genome alignment tools that allow for rearrangement^27^; however, technical variation may have played a role in the identification of 3 SNPs and the discrepancy in genome length between the two isolates.

This study confirms that *L. plantarum* ATCC 202195-B, which was only recently made commercially available, is genetically identical to the isolate of *L. plantarum* ATCC 202195 first deposited into the ATCC over 20 years ago and presumably identical to the isolate of *L. plantarum* ATCC 202195 used in the hospital and community-based trials conducted in India^5,7^. *L. plantarum* ATCC 202195 does not contain any unexpected AMR patterns and it is susceptible to multiple clinically important groups of antimicrobial agents. We show that *L. plantarum* ATCC 202195 contains two plasmids, but since there are no concerning plasmid-encoded antimicrobial or virulence genes, *L. plantarum* ATCC 202195 does not pose a material threat for the transfer of AMR or virulence factors to other microorganisms. While the probiotic potential of *L. plantarum* ATCC 202195 has been described in an initial clinical trial^5^, the genomic characteristics that were identified in the current work, including the identification of genes involved in stress responses, cellular adhesion, carbohydrate metabolism, and vitamin biosynthesis, provide further evidence in support of the probiotic properties of *L. plantarum* ATCC 202195 and shed light on potential mechanisms by which the strain exerts its biological effects on the human host. Taken together, the findings arising from this study will inform the design of future clinical trials and programs to employ *L. plantarum* ATCC 202195 for use as either a probiotic or, together with FOS, as a synbiotic^59^.

## Materials and methods

### Bacterial strains and isolates

*Lactiplantibacillus plantarum* ATCC 202195-A was procured from the ATCC (Manassas, Virginia) by International Flavors & Fragrances Inc. (formerly DuPont Nutrition & Biosciences) in October 2017. *L. plantarum* ATCC 202195-B was purchased from the ATCC by researchers at the Hospital for Sick Children (SickKids) in March 2019.

### Antimicrobial susceptibility testing

Resistance profiles of *L. plantarum* ATCC 202195-A and *L. plantarum* ATCC 202195-B were tested against the following 20 antibacterial agents: ciprofloxacin (0.5–2.0 µg/ml), erythromycin (0.25–4.0 µg/ml), meropenem (0.25–2.0 µg/ml), ceftriaxone (0.12–2.0 µg/ml), clindamycin (0.12–2.0 µg/ml), gentamicin (2.0–16.0 µg/ml), penicillin (0.06–8.0 µg/ml), ampicillin (0.12–16.0 µg/ml), chloramphenicol (1.0–32.0 µg/ml), tetracycline (2.0–16.0 µg/ml), levofloxacin (0.25–8.0 µg/ml), linezolid (0.50–8.0 µg/ml), piperacillin-tazobactam (8.0–128.0 µg/ml/4.0 µg/ml), vancomycin (1.0–128.0 µg/ml), quinupristin/dalfopristin (0.12–4.0 µg/ml), daptomycin (0.25–8.0 µg/ml), rifampin (0.50–4.0 µg/ml), trimethoprim/sulfamethoxazole (0.5–4.0 µg/ml/9.5–76.0 µg/ml), gatifloxacin (1.0–8.0 µg/ml) and oxacillin + 2% NaCl (0.25–8.0 µg/ml) using broth dilution assays in the Clinical Microbiology Laboratory at the Hospital for Sick Children (Toronto, ON, Canada).

Susceptibility testing was performed according to the Clinical and Laboratory Standards Institute (CLSI), M07, 11th edition and M45, 3rd edition, guideline for *Lactobacillus* spp.^19,60^. Direct colony suspensions, prepared to an equivalent of 0.5 McFarland standard in cation-adjusted Mueller–Hinton broth supplemented with lysed horse blood (CAMHB-LHB), were inoculated into three different commercially available Sensititre plates: Gram Positive MIC, Streptococcus STP6F AST and Gram Negative GN4F AST plates (ThermoFisher). Sensititre plates were incubated for 48 h at 35 °C, 5% CO_2_ and MICs were manually recorded and interpreted using clinical breakpoints established by CLSI^19^, the European Committee on Antimicrobial Susceptibility Testing (EUCAST)^20^ and the European Food Safety Authority (EFSA)^21^. Clinical breakpoint interpretations were categorized as susceptible ("S"), intermediate ("I"), resistant ("R"), no interpretive criteria available "("–”) and insufficient evidence ("IE"). To assess the potential for variability in observed MICs, all assays performed using the Sensititre Gram Positive MIC plate were repeated in triplicate for both *L. plantarum* ATCC 202195-A and *L. plantarum* ATCC 202195-B. To minimize bias during interpretation, laboratory technicians and analysts were blinded to the 'A' or 'B' identity of each isolate.

Resistance of *L. plantarum* ATCC 202195-B to eight of the 20 antimicrobial agents assayed using broth dilution assays (ciprofloxacin, erythromycin, ceftriaxone, gentamicin, penicillin, chloramphenicol, tetracycline and vancomycin) was also tested using gradient strip diffusion (Epsilometer test) at the Child Health Research Foundation (CHRF) in Dhaka, Bangladesh. E-strips were procured from AB Biodisk, Sweden (penicillin (0.002–32.0 µg/ml), ceftriaxone (0.002–32.0 µg/ml), gentamicin (0.016–256.0 µg/ml), tetracycline (0.016–256.0 µg/ml) and chloramphenicol (0.016–256.0 µg/ml), Hi Media, India [ciprofloxacin (0.002–32.0 µg/ml), erythromycin (0.016–256.0 µg/ml)] and Liofilchem, Italy (vancomycin (0.016–256.0 µg/ml). E-testing was performed as recommended by the CLSI^19^. In brief, a single colony of *L. plantarum* ATCC 202195-B was emulsified in normal saline to achieve turbidity to an equivalent of 0.5 McFarland standard. The inoculum was swabbed over the entire surface of a Mueller–Hinton agar plate supplemented with 5% sheep blood. After the plate had dried (approximately 10 min), E-strips were placed onto the surface of the agar. MICs, which were determined based on the edge where growth inhibition intersected the side of the E-strip, were recorded after incubation for ~ 24 h at 37 °C and then interpreted using breakpoints from CLSI^19^, EUCAST^20^ and EFSA^21^. MIC interpretations were categorized using the same criteria applied to the broth dilution assays. *L. plantarum* ATCC 202195-B was also tested for beta-lactamase activity using Nitrocefin (BD BBL, New Jersey, USA).

### Bacterial propagation and nucleic acid extraction

*Lactiplantibacillus plantarum* ATCC 202195-A was cultured anaerobically using the BD GasPak EZ container system (BD 260001) in 4 ml of Man-Rogosa-Sharpe (MRS) broth at 37 °C for 16–18 h. Genomic DNA was extracted using the DNeasy Blood and Tissue Kit (Qiagen, Model: 69506) following the pre-treatment protocol optimized for Gram-positive bacteria. In brief, 2 ml aliquots of *L. plantarum* ATCC 202195-A were centrifuged at 10,000×*g* for 5 min to harvest bacterial cells. Cells were resuspended in 180 µl of 1× Tris/EDTA buffer at pH 8.0 (BP2473-1, ThermoFisher) containing lysozyme (20 mg/ml) and then incubated for 30 min at 37 °C. Genomic DNA was eluted into 125 µl of elution buffer. RNA was removed by adding RNase (5 µg/µl) followed by incubation for 30 min at 37 °C. DNA yield and quality were assessed by electrophoresis in a 2% agarose gel and using a Qubit 2.0 fluorometer, respectively.

*L. plantarum* ATCC 202195-B was cultured in 5 ml MRS broth, which was incubated without shaking under aerobic conditions for 16–18 h at 37 °C and aerated with 5% CO_2_. Genomic DNA was purified using the One-4-All Genomic DNA Miniprep Kit (Bio Basic, Markham, Ontario, Canada) according to the manufacturer's protocol, with the addition of an enzymatic lysis step where bacterial cells were resuspended in lysis buffer (20 mM Tris–HCl, pH 8.0, 2 mM Na ETDA, 1.2% Triton X-100, 20 mg/ml lysozyme) and then incubated at 37 °C for 60 min. DNA yield and quality were assessed using a ratio of absorbances measured at 260 nm and 280 nm and 230 nm and 260 nm, respectively, using a NanoDrop 2000c spectrophotometer (ThermoFisher Scientific, Wilmington, MA).

### Plasmid DNA extraction

*Lactiplantibacillus plantarum* ATCC 202195-A was cultured in 5 ml MRS broth, which was incubated without shaking under aerobic conditions for 16–18 h at 37 °C and aerated with 5% CO_2_. Plasmid DNA was isolated using the QIAprep Spin Miniprep Kit (Qiagen, Germany), following the manufacturer's guidelines. Extracted plasmid DNA yield and quality was assessed by Nanodrop, as described above, and agarose gel electrophoresis.

### Whole genome sequencing and de novo genome assembly

Whole-genome shotgun sequencing of *L. plantarum* ATCC 202195-A was performed at the Roy J. Carver Biotechnology Center, University of Illinois Urbana-Champaign, using methods previously described^61^. In brief, Illumina reads were prepared using the Hyper library preparation kit (Kapa Biosystems, Roche, Basel, Switzerland) and sequenced on Illumina MiSeq with paired-end reads 250 nucleotides in length. For Oxford Nanopore (ONT) sequencing, 1 µg of DNA was sheared in a g-Tube and barcoded with 1D Native barcoding genomic DNA kit (EXP-NBD103 and SQK-LSK108). Ten libraries were pooled and sequenced on a GridION X5 sequencer. ONT reads were base-called using Albacore v. 2.1.10 (Oxford Nanopore Technologies, Oxford, United Kingdom). Raw paired-end reads were trimmed using Sickle (v1.3)^62^. Hybrid assembly of the Illumina and ONT reads was performed using Unicycler (v0.4.7)^63^, and the initial genome assembly was refined using Pilon^64^. To inspect the fidelity of the assembly, Illumina reads were mapped to assembled reference contigs using the Burrows-Wheeler Aligner (BWA) (v0.7.17)^65^ and Samtools (v1.9)^66^, and visualized using the Integrative Genomics Viewer (IGV) (v2.5.3)^67^. No manual corrections were made.

Whole-genome shotgun sequencing of *L. plantarum* ATCC 202195-B was performed at the Clinical Genomics Centre at Mount Sinai Hospital (Toronto, Ontario, Canada) using the NexteraXT platform (Illumina) with sequencing libraries prepared with the Nextera XT DNA library preparation kit. Paired-read sequence libraries were generated at 150 nucleotides in length, according to the manufacturer's instructions. Raw sequenced reads were trimmed using Trimmomatic^68^; nucleotides at the beginning and end of each sequencing read were discarded if the quality score was below 20 Phred. Reads shorter than 30 bases in length were also discarded. FastQ Screen^69^ was used to identify human genomic DNA contamination using PhiX as a calibration control. Reads associated with the human genome were removed from all subsequent analyses. BBNorm (v37.02)^70^ was used to normalize coverage in high-depth regions of the genome. SPAdes genome assembler (v3.13.3)^71^ was used to reconstruct the genome and the quality of the genome assembly was assessed using QUAST (v5.0.2)^72^.

Next generation sequencing of the extracted plasmid DNA from *L. plantarum* ATCC 202195-A was performed at the Clinical Genomics Centre at Mount Sinai Hospital (Toronto, Canada) using the same methodology employed for *L. plantarum* ATCC 202195-B whole genome sequencing. A stricter filtering and trimming protocol was utilized to process plasmid sequencing reads as per Gallegos^73^. In brief, raw reads were trimmed using Trimmomatic^68^, and discarded with a quality score below 30 and a minimum length of 50 base pairs. Trimmed reads were assembled using Unicycler (v0.4.7)^63^, with bold contig bridging. Assembled contigs were flagged as putative plasmid sequences if the assembled multiplicity was $\ge$ 10× or if the contigs were denoted as complete and circular. The resulting identified putative plasmid sequences were screened against the NCBI nucleotide database using BLASTn^31^ and PLSDB^74^.

### Comparative genome analysis

Overall similarity between the assembled genomes of *L. plantarum* ATCC 202195-A and *L. plantarum* ATCC 202195-B and other publicly available assembled genomes for *L. plantarum* ATCC 202195 (accession number: GCA_010586945.1 and GCA_004354995.1) were assessed by comparing the average nucleotide identities (ANI) using the Orthologous Average Nucleotide Identity Tool (OAT)^75^. A global alignment was performed using progressiveMauve^27^ and the contig mover feature was employed to account for variation introduced due to differing genome assembly tools used for *L. plantarum* ATCC 202195-A and *L. plantarum* ATCC 202195-B. The two bacterial genomes were then compared for SNPs. Paired-end normalized read libraries from the genome of *L. plantarum* ATCC 202195-B were aligned with the assembled genome of *L. plantarum* ATCC 202195-A using the BWA-MEM algorithm^65^ with default parameters. SNPs were identified using the variant caller FreeBayes (v1.2.0)^26^. For aligned reads to be evaluated as a potential variant, a minimum mapping quality of 20 was used and ploidy was set to 1. Filtering thresholds were employed to detect variants with a quality score > 10 and a read depth > 5. Variants that remained after filtering were assessed for their potential functional impact using SnpEFF^76^.

### Functional genome annotation of *L. plantarum* ATCC 202195-A and identification of antimicrobial resistance and virulence genes in *L. plantarum* ATCC 202195-A and *L. plantarum* ATCC 202195-B

The de novo assembled *L. plantarum* ATCC 202195-A genome was annotated using RASTtk (v1.3.0)^25^ and the evolutionary genealogy of genes: Non-supervised Orthologous Groups () Database^33^. Annotated genes were compared against the Clusters of Orthologous Groups (COG)^77^ and Kyoto Encyclopedia of Genes and Genomes (KEGG) databases^78^ to make inferences about higher order biological functions. Carbohydrate Active Enzymes (CAZymes) were identified using the meta server for automated carbohydrate-active enzyme annotation (dbCAN)^79^. CAZyme annotation of a putative *L. plantarum* ATCC 202195 gene was based on agreement between at least two of the three annotation tools employed by dbCAN, including HMMER which searched against the CAZy hidden Markov models database, DIAMOND which screened against the pre-annotated CAZY database, and Hotpep, which screened against a conserved CAZyme short peptide sequence database.

*Lactiplantibacillus plantarum* ATCC 202195-A was screened against five antimicrobial and virulence factor databases: Comprehensive Antibiotic Resistance Database (CARD)^28^, ResFinder^80^, Antibiotic Resistance Gene-ANNOTation (ARG-annot)^81^, Virulence Factor Database (VFDB)^29^ and the NCBI Bacterial antimicrobial resistance reference gene database using ABRicate (v0.5)^30,82^. Two different stringency thresholds for AMR gene detection were used: a high stringency threshold, which used a sequence identity and query coverage cut-offs of > 80% and a low stringency threshold employing a sequence identity cut-off of > 50% and a query coverage cut-off of > 10%. Targeted screening for resistance genes related to phenotypic resistance patterns was performed using BLASTp.

### Phylogenetic analyses

A total of 133 complete *L. plantarum* genomes were retrieved from NCBI in October 2020 (Accession numbers listed in Supplemental Table 2) and reannotated using Prokka (v1.14.5)^83^. The rapid large-scale prokaryote pan genome analysis (Roary) tool (v3.13.0)^84^ was used to identify the pan-genome of *L. plantarum* ATCC 202195-A and the other 133 *L. plantarum* genomes, including a previously submitted complete genome for *Lactiplantibacillus plantarum* ATCC 202195 (accession number: GCA_010586945.1), which was released in February 2020. The core pan-genome alignment was used to construct a phylogenetic tree with the maximum likelihood method using Randomized Axelerated Maximum Likelihood (RaxML) (v2.0.6)^85^ with fast bootstrapping, which was visualized using FigTree ^86^.

### Genome accession numbers

The sequence of *L. plantarum* ATCC 202195-A has been deposited in the GenBank database under accession number accession numbers CP063750.1, CP063751.1, and CP063752.1 (https://www.ncbi.nlm.nih.gov/nuccore/CP063750.1). The unassembled reads of *L. plantarum* ATCC 202195-B have also been deposited in the NCBI Sequence Read Archive (SRA) under the accession number SRR13686146.

## Supplementary Information

Supplementary Legend.

Supplementary Figure 1.

Supplementary Information.
