# Supplementary material for: Antimicrobial susceptibilities and comparative whole genome analysis of two isolates of the probiotic bacterium Lactiplantibacillus plantarum, strain ATCC 202195
Source: Sci Rep. 2021 Aug 5;11:15893. doi: 10.1038/s41598-021-94997-6 (PMC8342526; doi:10.1038/s41598-021-94997-6)
Supplement: Supplementary file 1 — Supplementary Legend. [file 41598_2021_94997_MOESM1_ESM.docx]

**Supplemental Figure 1.** Core-genome phylogenetic tree of the 134 strains of *L. plantarum*. The phylogenetic tree was generated using the alignment of the 1315 core genes, identified by the pan-genome analysis generated using Roary^82^. Maximum-likelihood calculations to determine phylogenetic relationships were performed by RAxML^83^ with fast bootstrapping. *L. plantarum* ATCC 202195-A is denoted as ATCC202195-A in blue. All other tip labels represent the assembly accession number for the remaining 133 *L. plantarum* strains. The phylogenetic tree was generated by Figtree (<http://tree.bio.ed.ac.uk/stats.html>) and edited in Inkscape v1.0.1 ([https://inkscape.org/release/inkscape-1.0.1/](https://urldefense.com/v3/__https:/inkscape.org/release/inkscape-1.0.1/__;!!D0zGoin7BXfl!r4fOpnIdOlACwwzaZ8YHNov8kVyXy7SRahvdd1yCXwIi_xvWrl7slSEzCZCeTZZq0sfR833YAf3Fvg$)). A complete list of assembly accession numbers and strain IDs can be found in **Supplemental Appendix Table 2**.
