# Supplementary figures and images for: Antimicrobial susceptibilities and comparative whole genome analysis of two isolates of the probiotic bacterium Lactiplantibacillus plantarum, strain ATCC 202195
Source: Sci Rep. 2021 Aug 5;11:15893. doi: 10.1038/s41598-021-94997-6 (PMC8342526; doi:10.1038/s41598-021-94997-6)

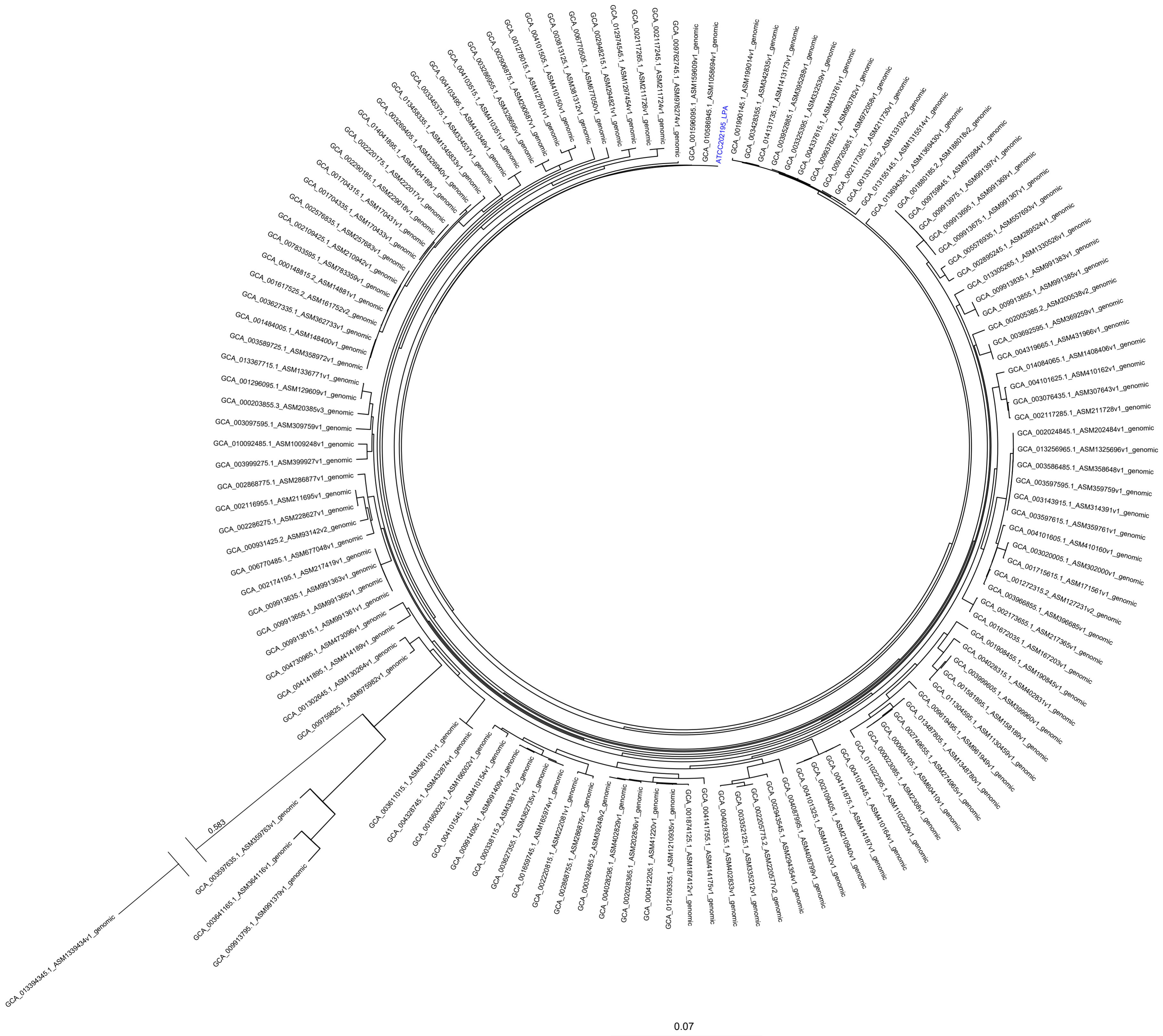

Supplemental Figure 1. Core-genome phylogenetic tree of the 134 strains of *L. plantarum*.

Supplement: Supplementary file 2 — Supplementary Figure 1. [file 41598_2021_94997_MOESM2_ESM.pdf]
